# Nature-based solutions extend the lifespan of a regional levee system under climate change

**DOI:** 10.1038/s41598-025-99762-7

**Published:** 2025-05-09

**Authors:** Rae Taylor-Burns, Borja G. Reguero, Patrick L. Barnard, Michael W. Beck

**Affiliations:** 1 Center for Coastal Climate Resilience, University of California, 1156 High Street, Santa Cruz, CA 95064 USA; 2 Pacific Coastal and Marine Science Center, United States Geological Survey, 2885 Mission Street, Santa Cruz, CA 95060 USA; 3 Coastal Science and Policy, University of California Santa Cruz, 115 McAllister Way, Santa Cruz, California 95060 United States

**Keywords:** Natural hazards, Civil engineering

## Abstract

Nature-based solutions are receiving increasing attention as a cost-effective climate adaptation strategy. Horizontal levees are nature-based adaptation solutions that include a sloping wetland habitat buffer fronting a levee. They can offer a hybrid solution to reinforce traditional levees in estuarine areas—plants on the horizontal levee can provide wave attenuation benefits as well as habitat benefits, but how the design of horizontal levees influences risk of levee failure remains unquantified. We use a hydrodynamic model, XBeach non-hydrostatic (XB-NH), to assess the stability and sustainability of existing levees and determine how hybrid nature-based climate adaptation measures can reduce the risk of overtopping on levees in San Francisco Bay. We compare overtopping rates in the existing levee system and in a variety of nature-based adaptation scenarios using a range of widths and slopes of horizontal levees to assess how horizontal levees perform in reducing risk of flooding, both with present day conditions and sea level rise. We show that climate change will challenge existing levee flood defenses in San Francisco Bay and increase the risk of overtopping, and that the nature-based solution of horizontal levees can meaningfully reduce risk of overtopping while simultaneously supporting marsh habitat. Flood risk reduction and habitat provision are both maximized with more gradually sloping and wider horizontal levee designs. Results show that the risk of overtopping can be reduced by up to 30% with horizontal levees. This analysis provides insight into horizontal levee design considerations and a methodological approach to adapt levees to prepare for climate change in urban wave-exposed estuaries. We show that horizontal levees can support preparation for the projected impacts of sea level rise (SLR) while simultaneously providing new intertidal wetland habitat.

## Introduction

Human caused climate change is raising sea levels and increasing the threat of coastal flooding globally, forcing coastal communities to develop adaptation strategies to mitigate future hazard risk. Traditional engineering solutions such as seawalls and levees have long been used to reduce flood risk. However, as mounting evidence demonstrates that coastal habitats can play an important role in attenuating waves and reducing storm surge^1–3^, management interest is shifting towards nature-based climate adaptation^4,5^.

Coastal management practices across San Francisco Bay (“the Bay”), a densely urbanized estuary in California, USA, exemplify this shift. The Bay relies heavily on gray infrastructure for flood protection today, with nearly 70% of the present-day Bay shoreline protected by levees and berms^6^. This regional levee system currently provides baseline flood protection for existing conditions, but without significant investment in existing infrastructure, this aging levee system will not provide sufficient protection for future SLR^7^. With current flood protection structures in place, San Francisco Bay will make up two-thirds of the California population and property at risk of exposure to SLR- and storm-driven flooding by 2100^8^. To protect the 400,000 people and $150 billion in property at risk in the Bay, a gray infrastructure investment of about $335 billion would be required^6^. Highlighting the shift towards using nature for climate adaptation, in 2016 Bay Area voters passed Measure AA, which uses a $12 per year parcel tax to restore wetlands in preparation for climate change^9^. Furthermore, over the past decade studies exploring how the region can incorporate nature-based coastal adaptation have proliferated^10–12^.

**Fig. 1:**
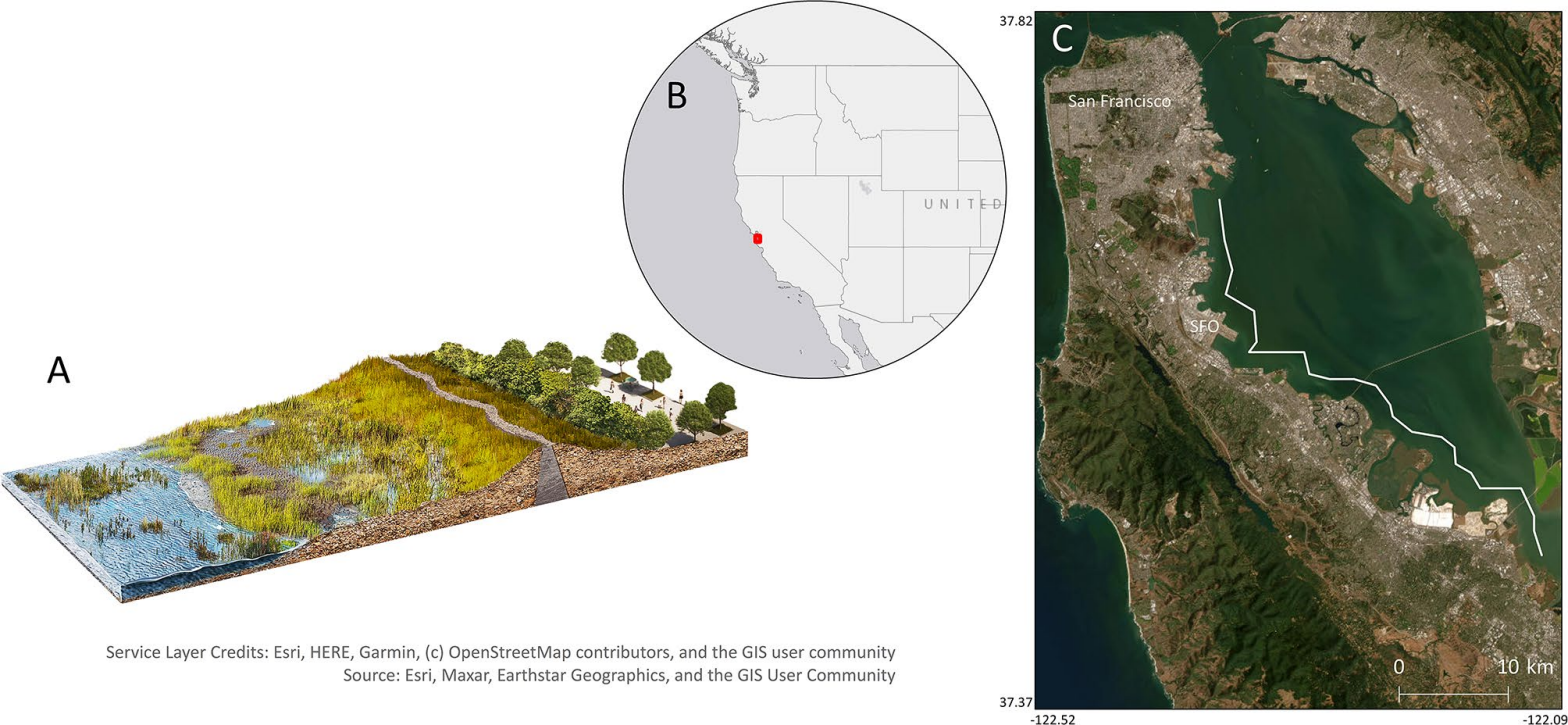
Depiction of a horizontal levee (**A**) and study location (**B** and **C**). Transects used in the hydrodynamic simulations are shown by the white lines in (**C**). Maps in **B** and **C** created with ArcMap 10.7.1 (https://desktop.arcgis.com/en/quick-start-guides/10.7/arcgis-desktop-quick-start-guide.htm).

Previous research suggests that vegetation can play an important role in decreasing the required height and associated cost of levees and that vegetation could reduce levee investment cost by $320 billion on a global scale^13^. Recent research also suggests that restoring marsh habitat in front of seawalls can be more cost effective in reducing flood risk than raising seawalls^14^. However, in highly altered urban estuaries such as San Francisco Bay (Fig. 1), where large areas of marsh habitat have already been reclaimed, developed, and degraded, large-scale habitat restoration options may be limited due to lack of accommodation space. An alternative approach to large-scale habitat restoration is the employment of vegetation in front of levees using horizontal levees (Fig. 1). Horizontal levees consist of implementing a levee set back from the coastline with a wide and gently sloping expanse of vegetated wetland habitat between the water and the levee. This configuration allows wave attenuation from plants to reduce the probability of wave-driven levee overtopping, while simultaneously supporting marsh habitat that can deliver other benefits. Horizontal levees have already been utilized for waste-water treatment purposes in San Francisco Bay and have been found to be effective in reducing concentrations of nutrients, pharmaceuticals, and other contaminants^15^. They can also support fish and wildlife and sequester carbon, all benefits associated with marsh habitat in the region^16,17^.

Horizontal levees have been identified as a potential flood risk reduction approach in San Francisco Bay based on ecological and geomorphological factors^10^. Recent work shows that vegetation can reduce risk of wave-overtopping on levees^13,18^, and regional studies identify suitable locations for horizontal levees in San Francisco Bay^10^. However, characterization of wave-resolved overtopping risk under present and future climate conditions is lacking, and how the design of horizontal levees influences risk of levee failure remains unquantified.

Here, we provide one of the first assessments to characterize how horizontal levees can serve as an effective option for nature-based adaptation in urban estuaries using San Mateo County in San Francisco Bay as a case study. To assess how horizontal levees can reduce the risk of wave-induced levee overtopping, we use a joint-probability analysis paired with a process-based numerical model, XBeach non-hydrostatic^19^. We test several horizontal levee designs across suitable regions of the Bay shore to quantify the capacity of this nature-based adaptation approach to reduce the risk of levee overtopping, at present and with climate change. We show that a hybrid nature-based climate adaptation approach can be a part of the solution in adapting to rising sea levels.

## Results

### Effects of sea level rise on overtopping risk

**Fig. 2:**
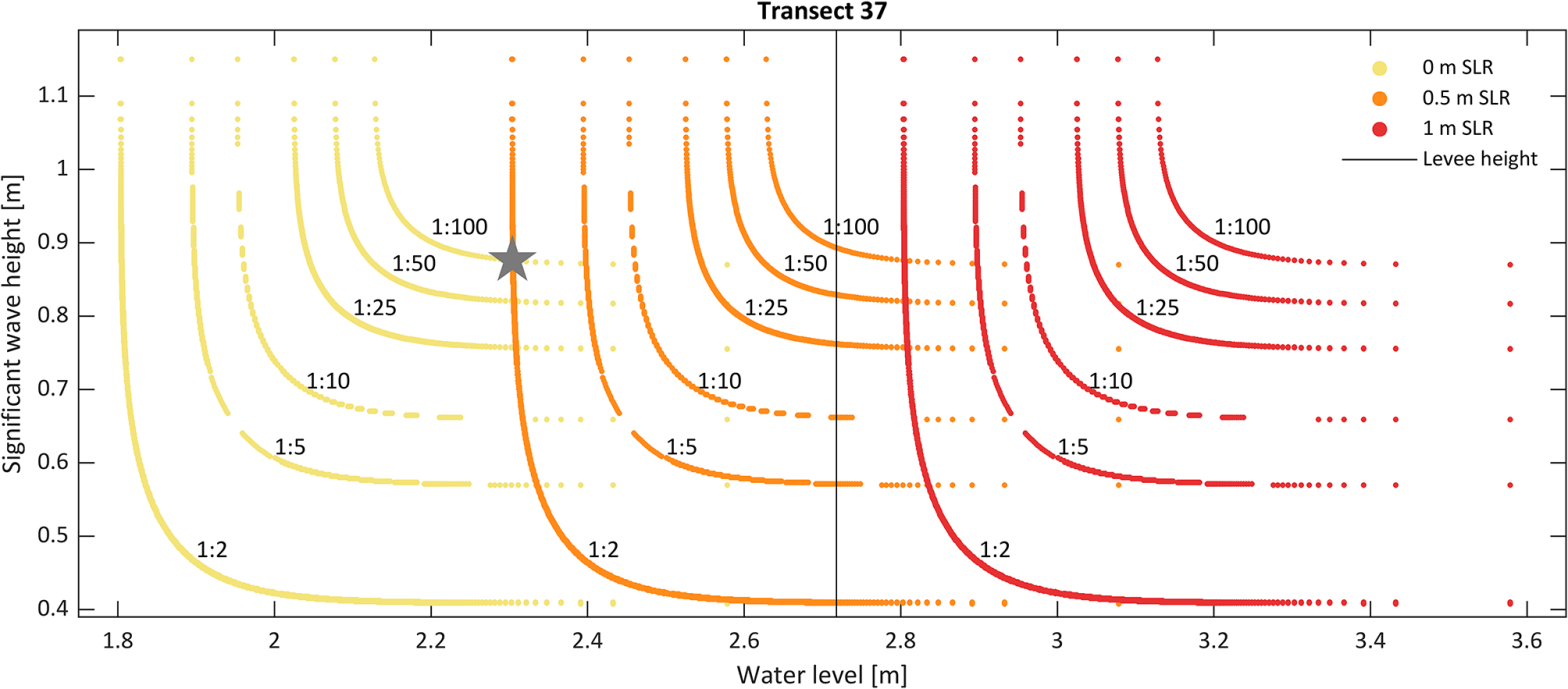
Joint probability return-period curves of co-occurring wave height and water levels on transect 37, with current sea level (yellow), 0.5 (orange) and 1 m of SLR (red). The black vertical line shows the levee crest height on transect 37. As sea levels rise, low probability events will become increasingly probable. An example of this increase in probability and frequency is shown by the gray star, which marks where with 0 m SLR 1:100-year event will cause the same wave and water level conditions as a 0.5 m SLR 1:2-year event. Thus, 0.5 m SLR makes 1:100-year storm conditions 50 times more probable.

Levees are commonly designed to withstand a certain low-probability event, such as a 1:100-year storm. The joint probability analysis illustrates that as sea levels rise, the design limits of these structures will be tested on an increasingly frequent basis. This is illustrated in Fig. 2, which highlights that as sea level rises, present-day low-probability events will become increasingly frequent. In Fig. 2, the probability of overtopping events is displaced linearly to the right with SLR, but event frequency increases non-linearly. For example, with 0.5 m SLR, which corresponds to the timeline of 2050–2075 according to the latest guidance for California^20^, the current 1:100-year wave and water levels will occur 50 times more frequently, with less than a 1:2-year probability. This increase in frequency is shown by the gray star in Fig. 2, which indicates that a 0 m SLR 1:100-year event will cause the same wave and water level conditions as a 0.5 m SLR 1:2-year event. These results highlight the urgent and growing need for adapting existing flood infrastructure. This analysis is conservative as it does not account for increases in wave height due to increased depth and fetch.

### Nature-based adaptation to climate change with horizontal levees

**Fig. 3:**
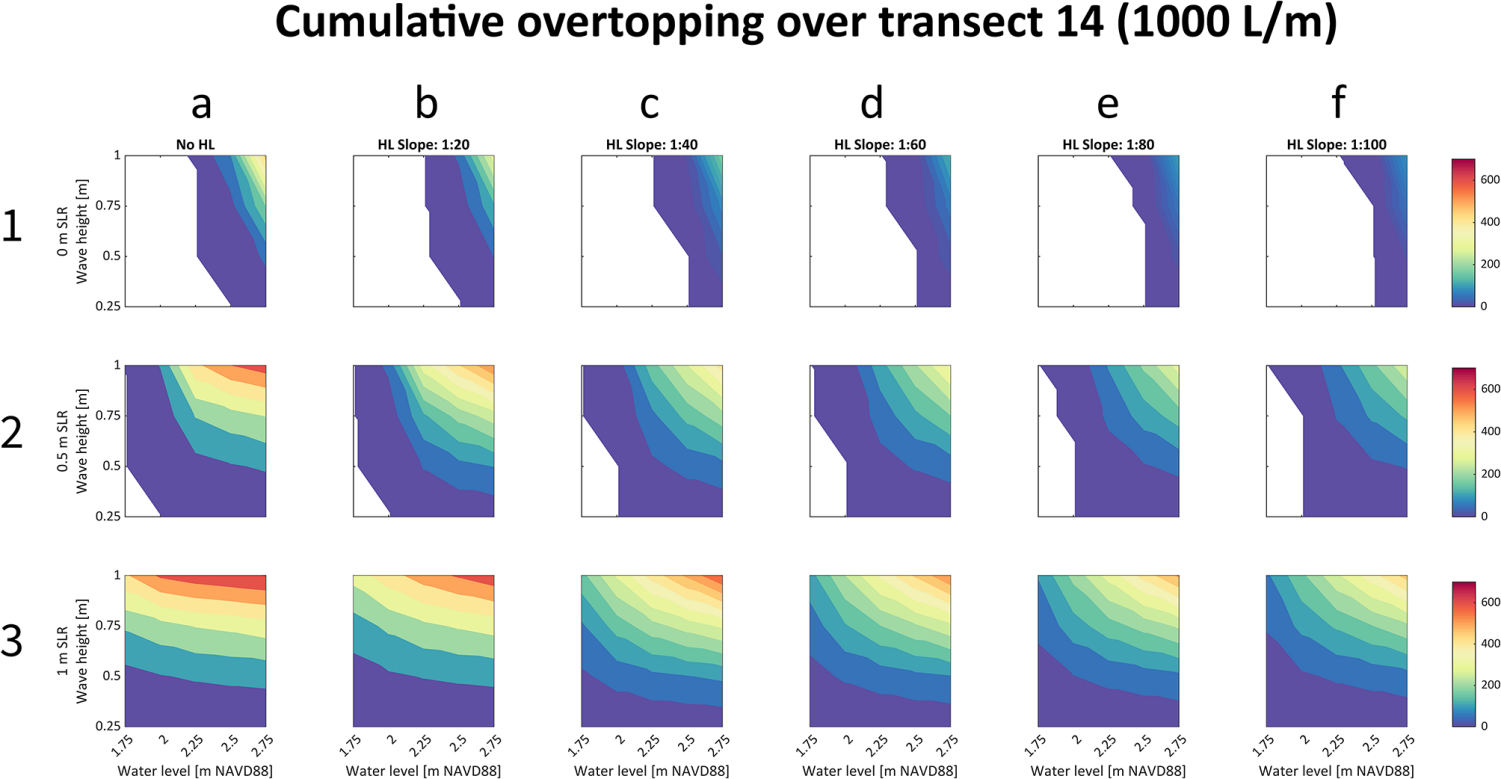
A comparison of the effectiveness of NBS adaptation options for reducing risk of levee overtopping and functional failure with sea level rise. using transect 14 as a representative case. Six different adaptation options are shown in each column (**a**–**f**); the rows (1–3) correspond to three different sea level scenarios: 0, 0.5 and 1 m. The color represents cumulative overtopping measured in 1000 L per meter (L/m). The colored areas in columns (**a**–**f**) show overtopping greater than 500 L/m, a critical threshold for functional levee failure. Expanding colored area and the increasing area of warm colors denote increased overtopping, as sea level increases going from top to bottom. As the slope of the horizontal levees decreases, the magnitude of overtopping decreases, as shown by the shrinking area of warm colors going from left to right in columns (**a**)–(**f**).

Established guidelines for overtopping thresholds for different types of coastal protection structures identify a peak flow of more than 10 L/m/s or a cumulative overtopping of more than 500 L/m/s as exceeding the design of levees like those in our study region^21^. These thresholds are used to identify XBeach simulations that exceed design thresholds and cause functional levee failure. The hydrodynamic analysis indicates that sea level rise will cause levee overtopping to become increasingly probable and severe. This can be seen in Fig. 3. Column 1 of Fig. 3 depicts overtopping across a single levee section in the study region under three SLR scenarios. Colored regions show where overtopping exceeds design thresholds under a range of surge and wave conditions, and warm colors show greatest magnitude of overtopping. As SLR increases (descending subplots in column 1), the colored area expands, illustrating that combinations of smaller waves and surge will exceed levee design thresholds into the future, thus increasing the probability of levee failure. Additionally, as SLR increases, the prevalence of warm colors increases, indicating that magnitude of levee overtopping will also increase into the future.

The hydrodynamic analysis also indicates that horizontal levees can reduce the risk of levee overtopping. This is also illustrated in Fig. 3. The columns of Fig. 3 depict overtopping across six different adaptation scenarios: an existing levee in column 1, and five horizontal levee scenarios with increasingly gradual slopes and increasing widths going from left to right in columns 2–6. Gradually sloping horizontal levees allow waves to decay over a longer distance and across more vegetation before they reach the levee and cause runup and overtopping. Moving from left to right in Fig. 3, as the slope of the horizontal levee becomes increasingly gradual and the width of the horizontal levee increases, the magnitude and probability of overtopping decrease. Decreasing magnitude of levee overtopping is shown by the shrinking area of warm colors going from left to right in columns (a)–(f) in Fig. 3. For example, with no adaptation (i.e. existing conditions), maximum cumulative overtopping in Fig. 3 is 475,000 L/m (Fig. 3, 1a), 654,000 L/m (2a), and 702,000 L/m (3a), with 0, 0.5, and 1 m of SLR, respectively. These values decrease to 377,000 L/m (1b), 550,000 L/m (2b), and 669,000 L/m (3b), with the implementation of a 1:20 horizontal levee and to 87,000 L/m (1f.), 288,000 L/m (2f.), 472,000 L/m (3f.) with the implementation of a 1:100 horizontal levee.

Additionally, as the slope of the horizontal levee decreases, the structural design threshold of the levee is surpassed in fewer simulations. This pattern indicates that more gradually sloping and wider horizontal levees are more effective at reducing risk of levee failure than steeper and narrower horizontal levees, due to increased frictional drag caused by a wider expanse of vegetation. This is shown in the top two rows of Fig. 3, in which the colored area shrinks from left to right, indicating that fewer surge and wave combinations exceed levee design thresholds as horizontal levee slope becomes more gradual. This effect is not present in the bottom row, which depicts results with 1 m of SLR. In this row the entire subplot is colored across all adaptation scenarios, suggesting that on this levee section, levee failure will occur with 1 m of SLR regardless of whether a horizontal levee is implemented. However, more gradually sloping levees will still reduce magnitude of overtopping with 1 m SLR, indicated by the shrinking area of warm colors as described above.

Simulations with high water levels and large waves result in the greatest cumulative overtopping but are also the least likely to occur (e.g. Fig. 2). In these low probability events, Fig. 3 illustrates that horizontal levees can reduce magnitude of overtopping, evidenced by shrinking areas of warm colors going left to right in the upper right corner of the subplots. In higher probability events, Fig. 3 illustrates that horizontal levees can reduce the risk of exceeding design thresholds, evidenced by shrinking areas of cool colors going left to right on the left and bottom side of the subplots. Thus, Fig. 3 indicates that horizontal levees can be effective in reducing the likelihood functional levee failure in low probability events and in reducing the magnitude of failure in higher probability events.

**Fig. 4:**
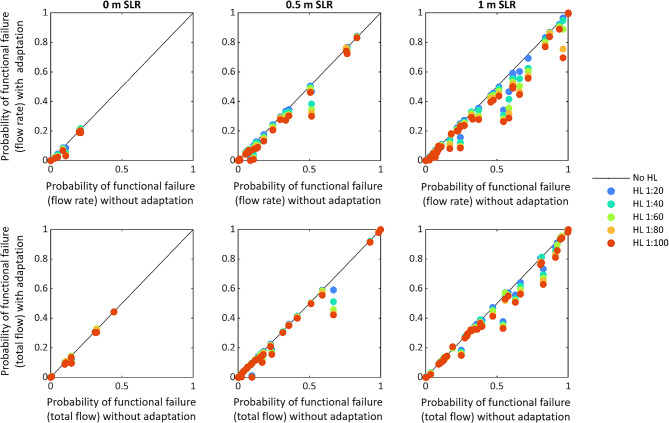
Functional levee failure risk reduction by nature-based adaptation. The probability of exceeding structural design thresholds of the levee is shown for all transects (48 in total) with five different horizontal levee designs. The top row shows probability of exceeding q > 10 L/m/s; and the bottom row shows probability of exceeding Q > 500 L/m. Each column shows a different sea level rise scenario. The color of the dots signifies the different slopes of the horizontal levee adaptations. Across the study region, the general trend shows that increasingly gradually sloped horizontal levees reduce the probability of exceeding the structural design thresholds. HL stands for horizontal levee in the legend.

The probability of exceeding levee design thresholds can be calculated by weighting the probability of occurrence of each event that exceeds thresholds and summing across the range of events. This is analogous to trapezoidal integration approaches that are used to determine annual expected damage across a range of storm return periods.^22^ Across all transects, the various horizontal levee designs influence the probability of exceeding two different structural design thresholds of the levee system. This is depicted in Fig. 4, which compares probability of exceeding design thresholds across the range of nature-based adaptation solutions tested.

Horizontal levees﻿ are ﻿generally effective in reducing risk, as indicated by the vertical distance between scattered data points and the diagonal black line in Fig. 4. More gradually sloping and wider horizontal levees reduce probability of exceeding structural design thresholds better than steeper sloped designs. This is indicated by the blue data points, which represent the steepest and narrowest horizontal levees, generally being closer to the diagonal black line than the red data points, which represent the most gradually sloping and widest horizontal levees. There are some locations where horizontal levees have limited influence on the risk of exceeding levee design thresholds, which is shown by the colored dots being clustered close to or on the diagonal black line. These instances are similar to the bottom row of Fig. 3, in which the levee design threshold is always surpassed with 1 m SLR regardless of horizontal levee implementation. There are also some locations where levee failure risk is zero and cannot be reduced by adaptation. These instances are depicted by points on the origin. However, most locations experience some degree of risk reduction by implementing horizontal levees. Because levees are increasingly overtopped throughout the study region with SLR, the risk reduction benefits of horizontal levees generally increase with SLR. The reduction in risk of exceeding design thresholds reaches up to 7%, 25%, and 30% with a 1:100 sloped horizontal levee, and up to 2%, 8% and 20% with a 1:20 sloped horizontal levee with 0, 0.5, and 1.0 m SLR respectively (Fig. 4).

**Fig. 5:**
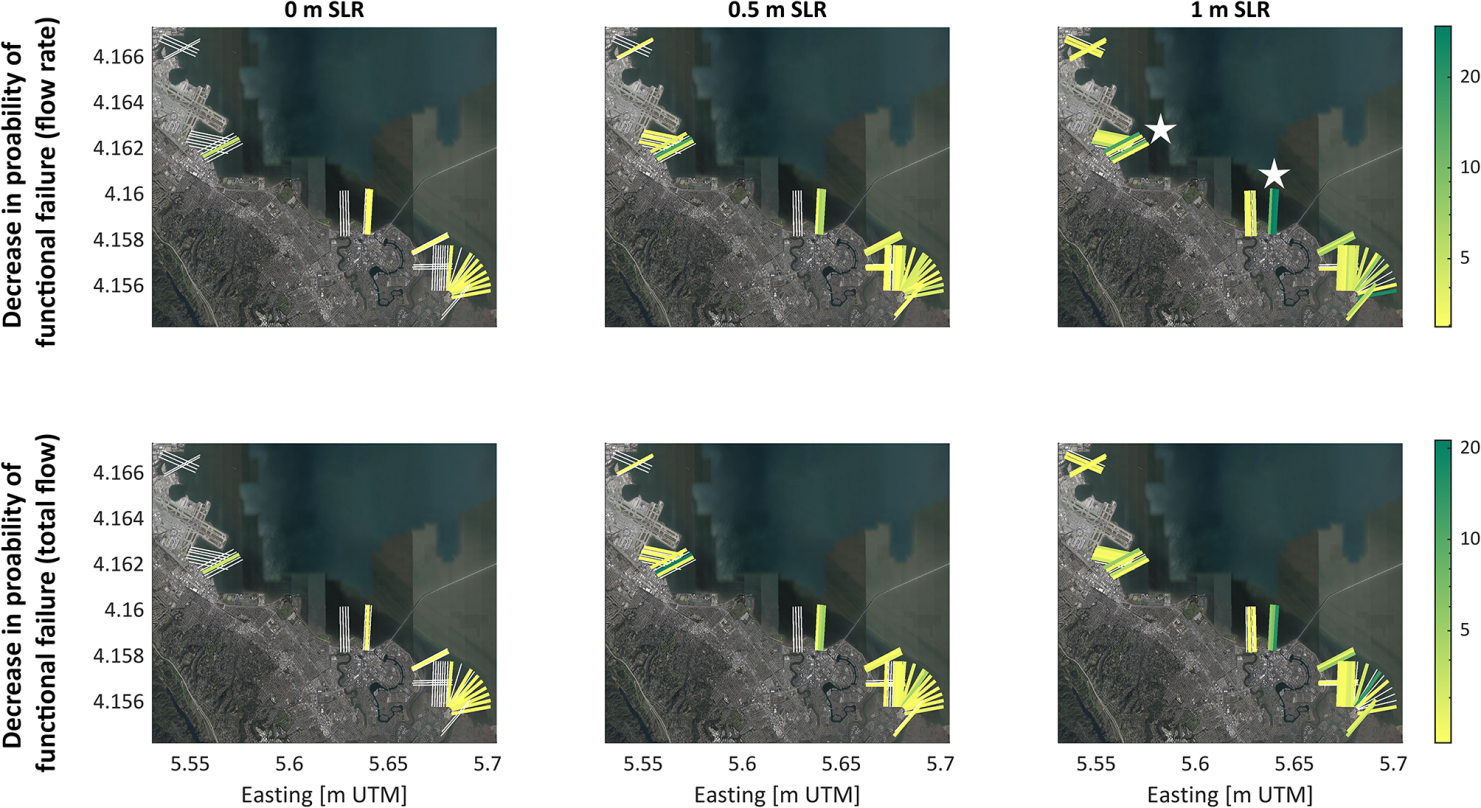
Comparison of decreases in probability of exceeding different levee design thresholds with horizontal levee adaptation. The color of each transect signifies the decrease in failure risk delivered by implementing a 1:100 sloping horizontal levee. The top row shows the decrease in risk of exceeding 10 L/m/s and the bottom row shows the decrease in risk of exceeding 500 L/m. The three different columns show three different sea level (SLR) scenarios. Green transects show places where horizontal levees are reducing the probability of levee failure by the greatest amount, reaching up to 30% in risk reduction. Horizontal levees play a stronger role as sea levels rise, shown by the increasing number of colored and dark colored transects, going from left to right. White stars in the top right plot denote hotspots of highest risk reduction from horizontal levees. Maps created with MATLAB 2023b (https://www.mathworks.com/products/new_products/release2023b.html) and basemap layer from Google Earth.

The spatial distribution of how much 1:100 sloped horizontal levees reduce risk of functional failure across the levee system of San Mateo County is shown in Fig. 5. In Fig. 5, white lines indicate levee sections where the risk of failure is zero, while the color on the thicker lines indicates the percent reduction in levee failure risk provided by horizontal levees, with green signifying the greatest magnitude of risk reduction. Risk reduction increases with SLR, depicted by the increasing prevalence of colored lines and the increasing number of green lines going left to right across the subplots. There are two main hotspots of risk reduction, indicated by the white stars in the top right subplot of Fig. 5. These two areas both have relatively deep waters directly offshore from the levee, with no existing marsh platform to attenuate waves. Implementing horizontal levees would provide the greatest risk reduction benefits in these locations.

While risk reduction varies widely across the region, mean values in reducing the risk of q > 10 L/m/s across all 48 transects are a reduction by 0.4%, 2.0% and 5.3% with 0, 0.5, and 1 m of SLR, respectively. Mean values in reducing the risk of Q > 500 L/m across all 48 transects are a reduction by 0.2%, 1.5% and 2.9% with 0, 0.5, and 1 m of SLR, respectively.

## Discussion and conclusions

We show that climate change induced sea level rise will challenge existing levee flood defenses in San Francisco Bay and increase the risk of exceeding design thresholds of the levee system, causing functional levee failure. We show that the nature-based solution of horizontal levees can reduce this risk while simultaneously supporting marsh habitat. Flood risk reduction and habitat provision are both maximized with gradually sloping and wider horizontal levee designs. The optimal slope will depend on the risk reduction desired, the wave and surge exposure, and the geomorphology of levee and coastal section.

SLR will challenge existing design thresholds and safety levels for flood defenses. This study provides one of the first insights into nature-based adaptation options to increase the resilience and performance of levee systems. Engineering performance metrics were employed to test the effectiveness of horizontal levees as a hybrid green-gray coastal climate adaptation solution. Results suggest that horizontal levees can be an effective approach to adapt to and mitigate the impacts of increasing flood risk in urban estuaries like San Francisco Bay. These results, like prior work, suggest that flood frequency will increase with SLR^23–25^ and that establishing horizontal levees in front of existing levee sections can reduce the risk of exceeding design limits of the levees. Although the study does not consider structural failure, overtopping can be linked to erosion and levee instability which can lead to breaching and catastrophic flooding. Vegetation-based adaptation can help reduce increased overtopping with SLR.

More gradually sloping and wider horizontal levees are better able to reduce risk of functional levee failure than narrower and steeper horizontal levees by providing a more expansive vegetation field that can more effectively reduce waves. For example, 1:100 sloped horizontal levees can reduce the risk of overtopping that exceeds the structural design of the levee by up to 30% with 1 m of SLR, while 1:20 sloped horizontal levees can reduce risk of structural exceedance by up to 20% with 1 m of SLR. However, there is a tradeoff between using more gradually sloping horizontal levees. Though they provide more risk reduction benefits, they will cost more to implement as they have a larger footprint, thus requiring a larger spatial area of construction and larger land purchases. Larger wetlands also provide more ecological, recreation, and carbon sequestration benefits, which offset some of these costs. Land purchase costs are non-trivial and are estimated comprise more than half the total cost of adapting the San Francisco Bay levee system to prepare for climate change^6^. Despite this tradeoff, meaningful risk reduction can be achieved with the steepest sloping horizontal levees tested in this study, which can provide up to 20% reduction in probability of functional levee failure (Figure S1). Furthermore, the cost of levee failure in urban areas is high—for example, in 2023 when a 400 ft section of a levee failed on the Pajaro River in Central California, damage totaled about $300 million, resulting in a cost of $750 thousand of damages per linear foot of failed levee^26^. This is approximately two orders of magnitude greater than the cost of constructing horizontal levees^27^, highlighting the fact that investments that reduce risk of levee failure could pay dividends in extreme events.

This work identified areas where horizontal levees would be most effective and found that horizontal levees will deliver the greatest risk reduction in areas where the current levee is fronted by relatively deeper water with little to no existing marsh platform. This information can help managers prioritize where to invest in horizontal levees, given limited adaptation funding.

Modeled wave decay in XB-NH has been calibrated in vegetated marshes^18^, but overtopping in XB-NH through vegetation remains uncalibrated. Overtopping in XB-NH has been compared to measurements and output from other models, with results suggesting that XB-NH has adequate skill in simulating overtopping despite limitations^28^. Lack of calibration may affect the uncertainty in these results but should not affect the interpretation of the comparative patterns identified here. Calibration and validation of overtopping through vegetation should be explored before horizontal levees are widely implemented in climate adaptation plans. Furthermore, while this study relied on a review equation relating the vegetation drag coefficient (C_d_) and the Reynolds number (Re), this relationship is still an area of active research. Thus, obtaining wave transformation and overtopping measurements across vegetation in high Reynolds number conditions should be a research priority. Even if these observations are obtained, it can be difficult to calculate C_d_ due to uncertainty and variation in methodology for vegetation surveys. This challenge has spurred recent research in quantifying wave attenuation in marshes by their standing biomass^29^, which requires only one field measurement rather than a minimum of three. Incorporating this approach into numerical models like XBeach would facilitate wave modeling through vegetation.

Sediment dynamics within marsh habitat are also important to consider. These dynamics control the degree to which marshes accrete sediment, which may or may not keep pace with SLR. San Francisco Bay marshes have been keeping up with SLR over the last 150 years by accreting 0.2–0.5 cm/year^16^, but these rates are less than 4% of what will be necessary to keep up with accelerating SLR by 2100^30,31^. Further exacerbating insufficient accretion rates, riverine flood control structures channel sediment out to open water and dams impound sediment upstream, both disrupting the historic connection between marshes and upland sediment sources. The relative elevations of marshes in San Francisco Bay are predicted to decrease by 0.4 to 1.3 m by 2100^32^, causing marshes that do not have migration space to transition to mudflats^33^. Sediment dynamics also influence whether marsh edge erosion or progradation will occur. Over recent decades, marshes in the wave-exposed northern part of San Francisco Bay have exhibited both spatial and temporal variability in edge change on a local scale^34^, though erosion is more likely to occur in a future with higher sea levels. Our model did not account for sediment interactions and future work could incorporate these processes.

This study assumed shore-normal flow by using one dimensional XB-NH, which was done due to computational limitations. This assumption is conservative because it ensures that, along most shorelines, waves take the shortest path across the marsh to the levee. However, it is possible that waves are focused on certain shoreline segments in embayments, and our approach neglects this possibility. Our approach also neglects the possibility of interactions between waves and alongshore currents.

This study does not cover structural failure that could be caused by overtopping flow. This study only focuses on functional failure from water flow over the levee crest. However, large overflows on the levee may lead to erosion and instability of the slope, leading to structural failure and breaching. This study also neglects the possibility of failure due to inertial forces introduced by high water levels and due to seismic activity. Six pathways for levee failure have been identified, including seepage and slumping/spreading, which can both be caused by high water levels without overtopping^35^. Studies in the Sacramento-San Joaquin Delta have assessed risk to that region’s levee system and show that failures can occur due to high water level events without overtopping and due to liquefaction caused by seismic activity^36,37^. Stability threats with higher sea levels may also be relevant in the Bay Area given that many parts of the Bay are built on fill (soft mud and sand), including several municipalities in San Mateo County. These areas are particularly vulnerable to liquefaction and many of them are within about 10 km from the San Andreas Fault. Further complicating these risks is the fact that most of the levees in San Francisco Bay are not certified by the Federal Emergency Management Agency and do not meet certain geotechnical engineering criteria, meaning their limits are not fully understood^54^. Thus, levees may fail for other reasons before overtopping ever occurs. A comprehensive geotechnical risk assessment for the Bay’s levee system would help illustrate how realistic this assumption is.

This is one of the first studies that illustrates the need of adapting flood defense systems in urban waterfronts and demonstrates that retrofitting with nature-based solutions can be an effective way to reduce risk as SLR progresses. Horizontal levees can meaningfully reduce risk of levee failure in urban estuaries like San Francisco Bay while simultaneously offering potential for other co-benefits such as habitat, improved water quality, carbon sequestration, and recreation opportunities. Further, the risk reduction benefits provided by horizontal levees increases with SLR, suggesting that with further study, this hybrid infrastructure approach can be a part of the solution as communities face the need to adapt to rising sea levels.

## Methods

### Study site

Within California, San Mateo County has the most projected flood exposure due to climate change, with more than 140,000 people and $50 billion of property at risk of flooding through this century^38^. San Mateo County’s bay shoreline is highly altered and contains critical public infrastructure, including the San Francisco International Airport, touchdowns of two regional bridges, an interstate highway, the heart of the Silicon Valley technology industry, and over 750,000 residents. Levees are an essential component of flood control throughout the region. San Mateo County is one of only six counties in the country with over 100,000 residents at risk of high-tide flooding with 0.9 m of SLR and assumed population growth^39^. A wide variety of pathways for integrating nature-based shorelines has been identified regionally^10,40,41^, including horizontal levees, which are also sometimes called ecotone levees. With the highest future flood risk in the state and stakeholder interest in incorporating nature into climate adaptation approaches, San Mateo County’s shoreline is a key study site for investigating the potential of horizontal levees as a nature-based flood defense.

### Numerical modeling

Effects of vegetation and levee overtopping are modeled with XBeach^42^, a process-based model which simulates nearshore processes including wave transformation, wave-induced setup, over-wash, and inundation. Recently, a non-hydrostatic mode (XB-NH) was developed for XBeach^19^, which is similar to a depth-averaged version of the SWASH model^43,44^ and is able to fully resolve incident-band (short period) waves. Resolution of incident-band waves, though computationally costly, is necessary to determine swash on relatively steep slopes and is thus necessary to investigate runup (as determined by the last wet grid cell), and potential overtopping on levees. Recent work has demonstrated the application of XB-NH for overtopping problems^28^.

Runup and overtopping in the non-hydrostatic mode have been validated using several separate datasets from sandy beaches. Results show that incident-band driven runup height is predicted with good accuracy and a maximum deviation from observations of 15%^45^. A reduced two-layer non-hydrostatic formulation is included and allows for a more accurate description of the frequency dispersion in relatively deeper water. Relative depth (KD) determines whether waves are in deep water (waves do not interact with the bottom, KD > π) or shallow water (waves interact with the bottom, KD < π/10), and is based on water depth and wavelength. Good performance has been established up to a relative depth of 3^19^.

XB-NH also allows for the inclusion of the effect of vegetation within the model via vegetation parameters including stem density, frictional drag coefficient, stem height, and stem diameter^46,47^. The combination of XB-NH and the vegetation module has been calibrated to wave attenuation data from San Francisco Bay^18^. XB-NH can be computationally costly, particularly in shortwave environments where wavelengths and grid cell sizes are small. As a result, XB-NH was applied in this study in one-dimensional mode along transects as opposed to in two-dimensional mode on a lateral grid, since the flow at the levee section is considered predominantly shore normal. This approach assumes that wave forcing is shore-normal and neglects lateral flow, but allows application of the tool to a larger area and in multiple scenarios.

The XB-NH set up included a grid spacing of 30 grid points per wavelength (total number of grid points ranged from 2,000 to 12,000). Sensitivity analyses showed that the model results converge at this resolution. A Manning’s friction formulation of 0.018 m^3^/s was used for the mudflat on the unvegetated portion of each transect. Simulations all included 10 minutes of spin-up which were not used in the analysis and were 1000 waves in duration.

### Transect development

In prioritizing where to apply one dimensional XB-NH within the study domain, both wave exposure and horizontal levee viability were considered. The areas of the San Francisco Bay shoreline that are suitable for horizontal levees have been identified, considering the appropriate elevation to support marsh habitat, proximity to urban development, and the capacity to accommodate a levee with at least a 1:30 slope^10^. To find sections of the San Mateo County shoreline that are more exposed to waves, wave heights and water levels were extracted from storm simulations^48^ approximately 120 m offshore from the first line of coastal defense. Wave formation in San Francisco Bay is limited by the Bay’s shallow depths and constrained area, but in the main channels of the Bay wave heights can exceed 1 m^49^. These waves can propagate towards exposed shorelines of the Bay. Coastal sections dominated by wind wave-levee interaction processes, as opposed to long-wave storm tide dominated areas, were identified through a ratio of wave and storm contribution to water level (shown in Eq. 1, where Hs is significant wave height and TWL is total water level). Areas with a mean relative wave height to surge ratio of greater than one which coincided with areas identified by the San Francisco Estuary Institute^10^ were selected for casting XB-NH transects (Figure S2).

1 $$Relative\, wave \,height \,to \,surge \,ratio =(Hs/(median \,Hs))/(\text{T}WL/(median \,TWL))$$

The Digital Shoreline Assessment System^50^ was used to cast transects and the results were modified manually to ensure transects are perpendicular to the existing levee. Bathymetry was interpolated using two different digital elevation models (DEMs): a 5 m resolution vegetation-corrected DEM, only available above -1 m NAVD88^51^ was used above + 1 m NAVD88 and a 10 m resolution DEM^52^ was used below + 1 m NAVD88. Switching between the two DEMs at + 1 m NAVD88 provided the smoothest transition.

Throughout our study region, mean sea level is about + 1 m NAVD88. 0.86—2.38 m NAVD88 was classified as marsh^53^. Vegetation stem density, diameter, and height for the marsh zone was taken from a meta-analysis^17^. Several regional vegetation surveys have been done in San Francisco Bay, but they do not resolve the frictional coefficient of vegetation (C_d_) across as wide a range of hydrodynamic conditions as the meta-analysis^17^. Because C_d_ is a key parameter in assessing wave transformation across vegetation, the meta-analysis including numerous marsh plant species was utilized rather than local surveys. Stem density was 500 stems/m^2^, stem height was 150 cm, and stem diameter was 7 mm. There are many areas in the study region that are at suitable elevation for marsh habitat but are developed and unvegetated. To ensure these areas did not get assigned as marsh habitat, vegetation was restricted to be offshore from first line of defense. The first line of defense was determined by spatial data published by the San Francisco Estuary Institute^54^. Numerous studies show that C_d_ has an inverse relationship with Reynolds number (Re), which increases with increasing turbulence. This inverse relationship is caused by the bending and flexing of plant stems, which create less frictional drag in turbulent conditions. This relationship is important to account for when simulating wave decay by plants^55^ to avoid overestimation of frictional drag by plant stems^17^. A generalized relationship between Re and C_d_ in marsh plants has been developed^17^, which is based on uniformly post-processed data from 14 different studies ranging up to Re < 3000 (Eq. 2):

2 $${\mathit{log}}_{10}\left({{\varvec{C}}}_{{\varvec{d}}}\right)= \left(-1.72 \pm 0.93\right)+\left(-1.67 \pm 1.19\right)\times {\mathit{log}}_{10}\left(3 \times {10}^{-4}\times {\varvec{R}}{\varvec{e}}\right)$$

Equation 2 was used to determine appropriate drag coefficients for the marsh vegetation in the XB-NH model. An initial batch of simulations was completed across the full range of water level and wave combinations derived from the joint probability analysis. This was done to determine Re at the offshore edge of the vegetated zone within each transect. Re was then used to determine appropriate C_d_ for the vegetation within the marsh, as shown in Figure S3. Experimental simulations were conducted with the C_d_ value calculated from Eq. 2.

### Wave and sea level data

Two previously modeled hindcasts were used to develop boundary conditions of water level and wave height for this model. A 30-year hindcast simulated wave heights in San Francisco Bay using global climate model of near-surface daily winds paired with observations from four regional airports^49^. This approach allowed the authors to keep the spatial resolution of modeled winds while increasing the temporal resolution by temporally downscaling and using observations to find a “best match” day. The maximum over-water windspeed was extracted each day of the hindcast and used to force a SWAN (Simulating WAves Nearshore) model, resulting in a 30-year hindcast of daily maximum wave heights across San Francisco Bay. A 70-year hindcast used Delft3D Flexible Mesh to simulate water levels in San Francisco Bay from 1950 to 2019.^56^ Tidal constituents and a remote non-tidal residual (NTR, signal minus tide) forced the model at the open boundary. ERA5 wind and atmospheric pressure data^57^ forced the model at the surface boundary. Creeks and delta flows were included in the model. Upstream boundaries for creeks were forced with data from 16 river discharge stations from the USGS. Upstream boundaries for the Delta were forced with estimates of Dayflow, which is an estimate of the daily average outflow from the Delta, accounting for water consumption across the region.^58^ Modeled data was compared to observations from 15 NOAA tide gauges across the region, with an average skill score of 8 cm root mean square error. Point observations (model output time series at given locations) are publicly available at about 500 m increments along the coast of San Francisco Bay. The resulting values from the hindcast are representative of mean sea level, but because this study aims to assess levee overtopping, which is most likely to occur at high tides, a constant value of 0.85 m was added to the water level time series, which is representative of the difference between MHHW and MSL in this study region^53^.

Boundary conditions were derived using a joint probability analysis toolbox called MvCAT^59^, which requires time series observations of two variables at equal time steps. Thus, hindcasted wave conditions and water levels from the overlapping 30-year time-period of 1974–2004 were used for the joint probability analysis. Daily wave height values were extracted from the 30-year wave hindcast at the end of each transect. Total water level data were extracted from the 70-year total water hindcast at the corresponding times (00:00 each day) from the nearest model output point to the offshore end of each transect and the tidal signal was removed using a low pass filter. The toolbox includes 26 different copula and was used to determine joint probability curves for each transect. Across all 48 transects, the Gaussian copula had the overall best score, so this copula was selected for determining return interval curves. Ranking for the various copula are shown in Table S1.

Wave heights and water levels at 25 cm increments and spanning the domain of the return interval curves values produced from the joint probability analysis were used as boundary conditions for the XB-NH simulations. The return period was interpolated across the two-dimensional space, thus assigning a return period for each combination of wave height and water level boundary conditions used as boundary conditions. An example of this approach is shown in Figure S4, which shows hindcast wave and water level data in small gray circles. These points were used as input to the joint probability analysis, which produced the return period curves, shown in the thick black lines. The return period curves were then used to interpolate probability onto water level and wave combinations, shown in the large colored dots. The large colored dots were used as boundary conditions for XBeach simulations. Some of these boundary conditions are outside the range of modeled wave height^49^ and water level^56^. The interpolated return period of these simulations is more uncertain than values that are within the range of the modeled time series. This uncertainty was quantified by bootstrapping with resampling. 1000 samples with a length of the full 30-year time series (10,950 samples) were randomly taken from the original time series with replacement, and the MvCAT toolbox was used on each of the 1000 samples to create bootstrapped return period curves. These are depicted by the gray lines in Figure S4, and they highlight that uncertainty in return period curves increases with higher return periods. The subsequent analyses use the original return period curves, which are shown in black. The joint probability analysis was used to assess how return period probabilities for combinations of wave heights and water levels will change with SLR.

### Horizontal levee design alternatives

To assess how horizontal levee configuration influences wave decay and overtopping of the levee system, a range of slopes was applied to change bathymetry offshore from the last vegetated grid cell, representing the shoreward edge of the marsh and the location of the existing levee. Slopes tested included 1:20, which is the steepest recommended slope^10^, 1:40, 1:60, 1:80, and 1:100. More gradual slopes result in a wider area of marsh habitat supported on the seaward side of the levee. While the width of the different slopes varied based on the height of the existing levee and bathymetry, the 1:100 designs generally had a width five times greater than the 1:20 designs. Vegetation zones were shifted accordingly, based on changes in elevation, so that the area between 0.86 and 2.38 m NAVD88 was classified as marsh in each scenario.

The sum of all shoreward flow over the levee crest was calculated for each simulation to determine cumulative flow over the levee crest, representing levee overtopping during the peak of a storm as it coincides with high tide. Each simulation was 1000 waves long, so the total time over which cumulative overtopping was calculated varied based on wavelength, but was generally between 30 and 90 min, a period short enough to coincide with the peak high tide, during which overtopping is most likely. Cumulative flow over the levee crest and flow rate over the levee crest were compared to structural design thresholds identified by the EurOtop Manual^21^ as shown in Table S2. The EurOtop Manual is a commonly used and widely cited document which has been applied and updated over decades, providing guidance on analysis and prediction of wave overtopping on various flood defenses.

## Supplementary Information

Supplementary Information.

## Data Availability

All data and code are available upon request from corresponding author.
