# Supplementary material for: Nature-based solutions extend the lifespan of a regional levee system under climate change
Source: Sci Rep. 2025 May 9;15:16218. doi: 10.1038/s41598-025-99762-7 (PMC12064823; doi:10.1038/s41598-025-99762-7)
Supplement: Supplementary file 1 — Supplementary Information. [file 41598_2025_99762_MOESM1_ESM.docx]

## Supplemental material

Any use of trade, firm, or product names is for descriptive purposes only and does not imply endorsement by the U.S. Government.


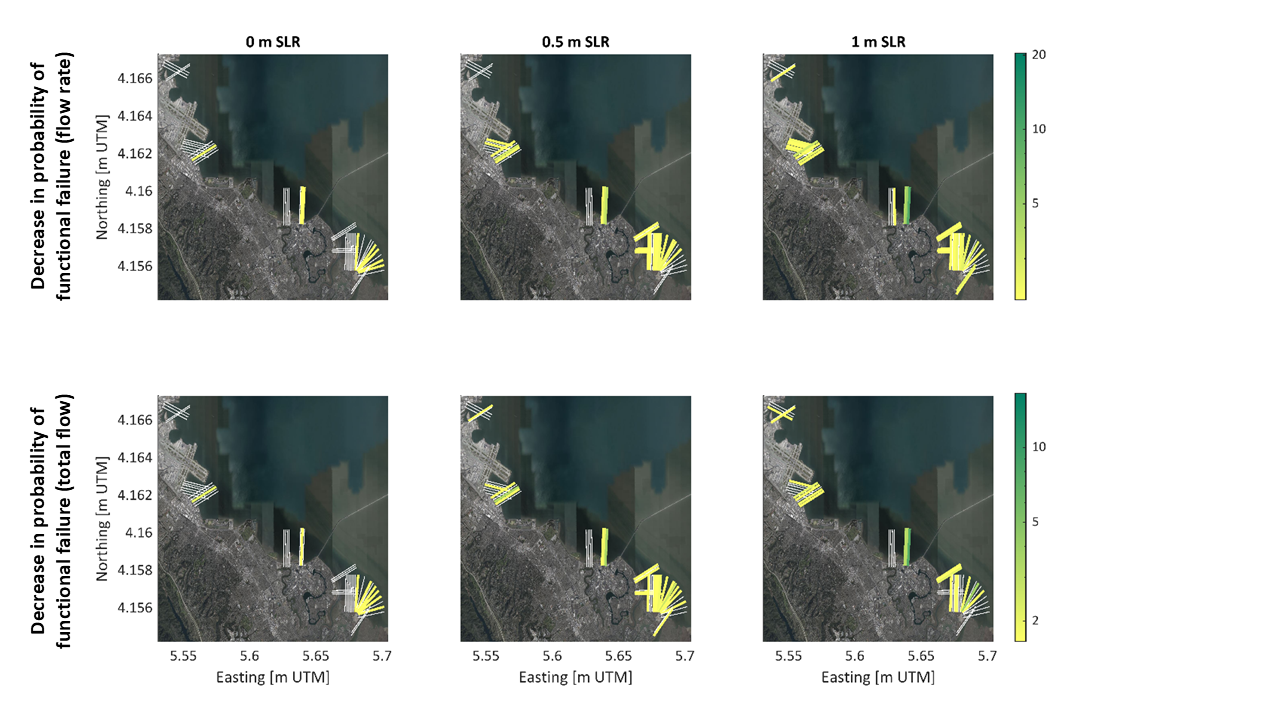


Figure S1. Comparison of decreases in probability of exceeding different levee design thresholds with horizontal levee adaptation. The color of each transect signifies the decrease in failure risk delivered by implementing a 1:20 sloping horizontal levee. The top row shows the decrease in risk of exceeding 10 L/m/s and the bottom row shows the decrease in risk of exceeding 500 L/m. The three different columns show three different sea level (SLR) scenarios. Green transects show places where horizontal levees are reducing the probability of levee failure by the greatest amount, reaching up to 20% in reduction of failure risk.


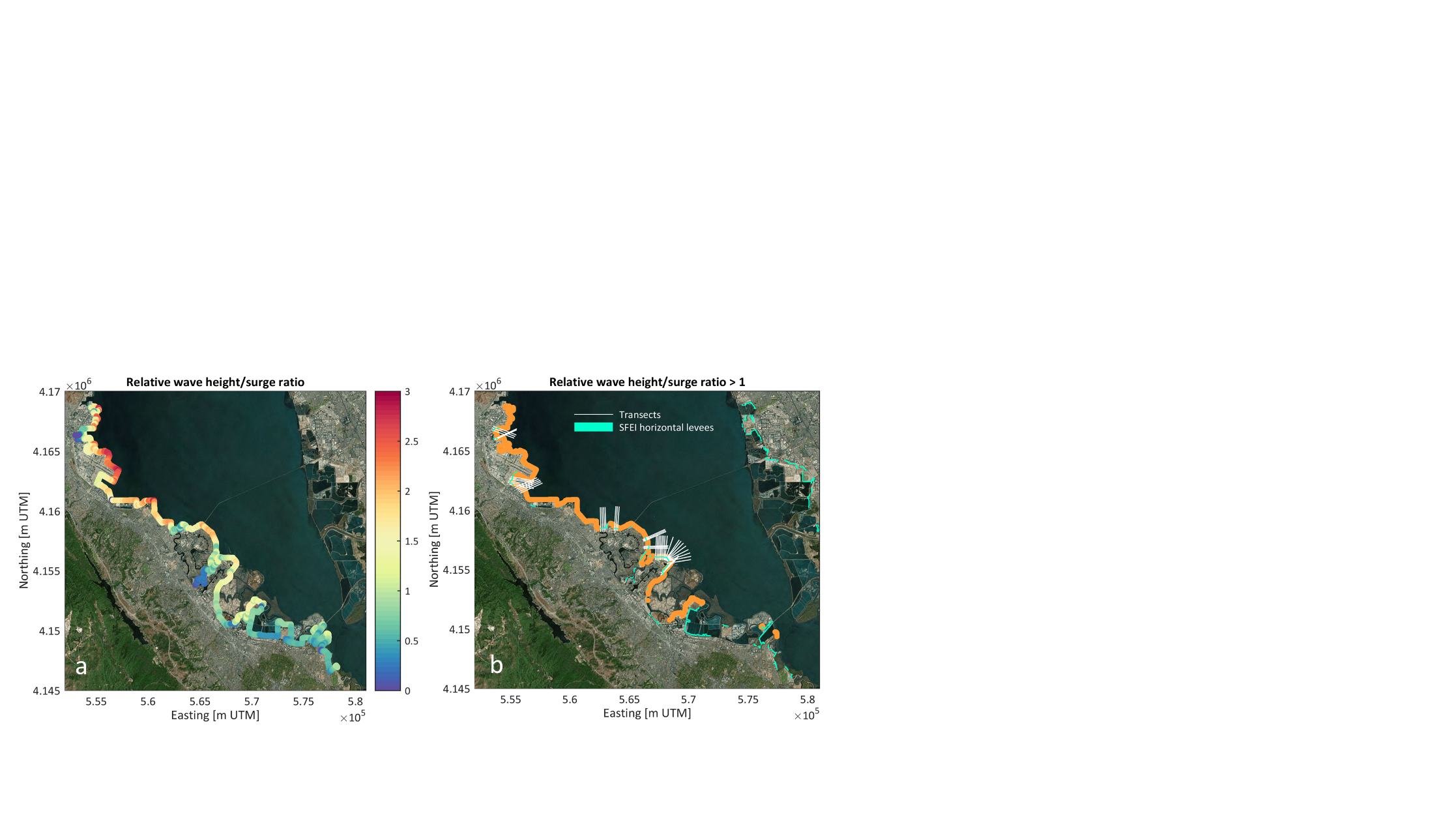


Figure S2. The relative wave height to surge ratio along throughout the study region (A) and areas with a relative wave height to surge ratio shown in orange, areas identified (Beagle et al. 2019) as suitable for horizontal levees shown in teal and transects used in this study shown in white (B).


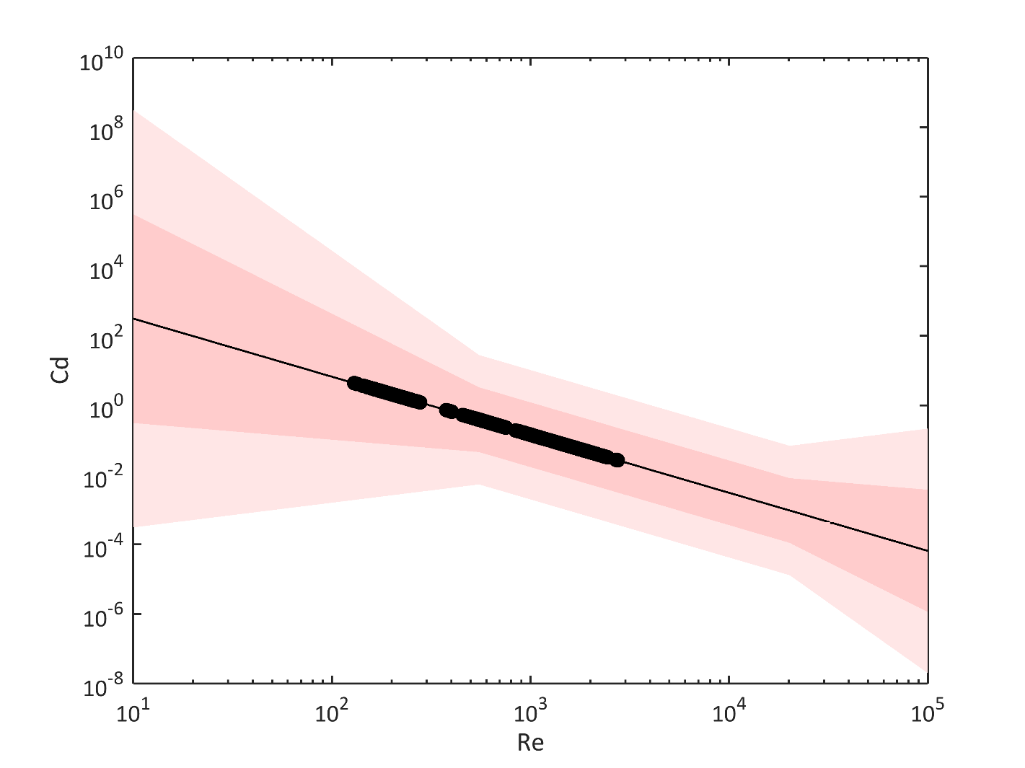


Figure S3. A plot of the Pinsky et al. (2013) equation relating vegetation drag coefficient (Cd) and Reynolds number (Re) in marshes. The equation is shown on the black line, with one and two standard deviations shown in dark and light pink, respectively. The black dots represent conditions in our experimental runs.


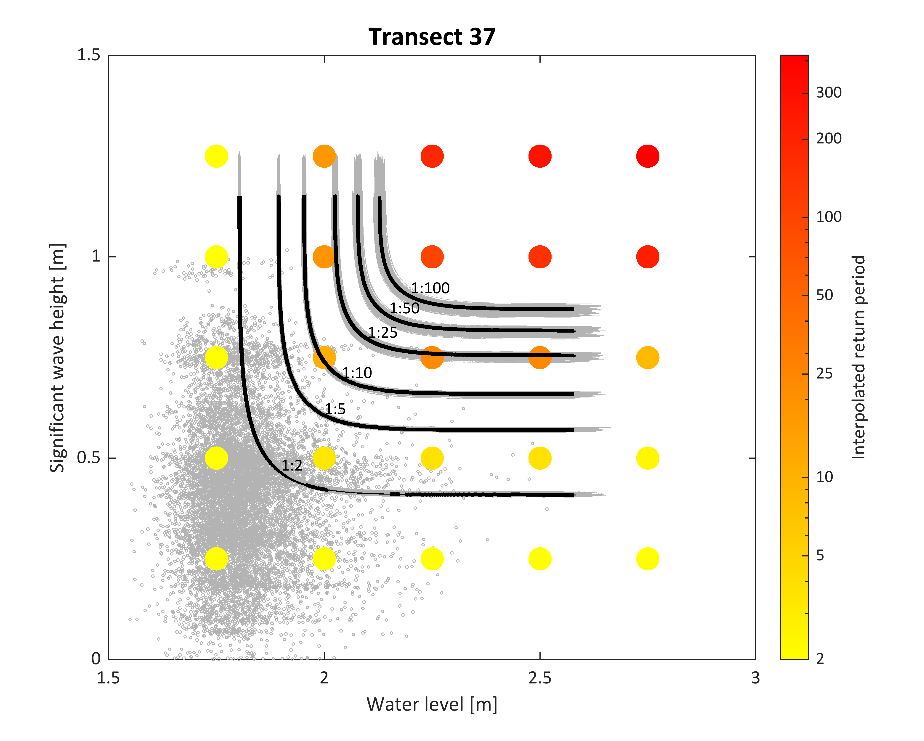


Figure S4. Gray dots show hindcast wave height (O’Neill et al., 2017) and water levels (Nederhoff et al., 2021) at or near the end of transect 37. These values were used as input for the joint probability analysis, which produced the return period curves which are shown in black. Text labels signify what the return period is for each of the six black lines. The gray lines underneath the thick black lines represent 1000 bootstrapped curves for each return period. The return period curves were used to determine the range and probability of boundary conditions in XB-NH. The boundary condition wave and water levels for XB-NH simulations are shown by the large colored dots. Color of the large dots represents interpolated return period for the water level and wave height combination. Yellow indicates short return period, or high probability, and red indicates long return period, or low probability. Thus, high water levels paired with large waves are less probable (red) than low water levels and with smaller waves (yellow).

Table S1. Top five copula rankings for each of the 48 transects.

|  | Top 5 Copula Ranking | | | | |
| --- | --- | --- | --- | --- | --- |
| ***Transect*** | **1** | **2** | **3** | **4** | **5** |
| *1* | AMH | t | Gaussian | FGM | Fischer-Kock |
| *2* | AMH | t | Gaussian | FGM | Fischer-Kock |
| *3* | AMH | t | Gaussian | FGM | Fischer-Kock |
| *4* | AMH | Gaussian | t | FGM | Fischer-Kock |
| *5* | AMH | Gaussian | t | FGM | Fischer-Kock |
| *6* | AMH | Shih-Louis | Gaussian | FGM | Fischer-Kock |
| *7* | AMH | Gaussian | FGM | Fischer-Kock | t |
| *8* | AMH | Gaussian | FGM | Fischer-Kock | t |
| *9* | AMH | Gaussian | FGM | Fischer-Kock | t |
| *10* | AMH | Gaussian | FGM | Fischer-Kock | t |
| *11* | AMH | Gaussian | t | FGM | Fischer-Kock |
| *12* | AMH | Gaussian | FGM | Fischer-Kock | t |
| *13* | AMH | Gaussian | t | FGM | Fischer-Kock |
| *14* | AMH | Gaussian | t | FGM | Fischer-Kock |
| *15* | AMH | Gaussian | t | FGM | Fischer-Kock |
| *16* | AMH | Gaussian | t | FGM | Fischer-Kock |
| *17* | Shih-Louis | Gaussian | AMH | t | FGM |
| *18* | Shih-Louis | Gaussian | AMH | t | FGM |
| *19* | Shih-Louis | Gaussian | AMH | t | FGM |
| *20* | Shih-Louis | Gaussian | AMH | t | FGM |
| *21* | Shih-Louis | Gaussian | Fischer-Hinzman | AMH | FGM |
| *22* | Shih-Louis | Gaussian | Fischer-Hinzman | AMH | FGM |
| *23* | Shih-Louis | Gaussian | Fischer-Hinzman | AMH | FGM |
| *24* | Shih-Louis | Gaussian | AMH | FGM | Fischer-Kock |
| *25* | Shih-Louis | Gaussian | AMH | FGM | Fischer-Kock |
| *26* | Shih-Louis | Gaussian | AMH | FGM | Fischer-Kock |
| *27* | Shih-Louis | Gaussian | AMH | t | FGM |
| *28* | Shih-Louis | Gaussian | AMH | t | FGM |
| *29* | Shih-Louis | Gaussian | AMH | t | FGM |
| *30* | Shih-Louis | Gaussian | AMH | t | FGM |
| *31* | Shih-Louis | Gaussian | AMH | t | FGM |
| *32* | Shih-Louis | Gaussian | AMH | t | FGM |
| *33* | Shih-Louis | Gaussian | AMH | FGM | Fischer-Kock |
| *34* | Shih-Louis | Gaussian | AMH | FGM | Fischer-Kock |
| *35* | Shih-Louis | Gaussian | AMH | FGM | Fischer-Kock |
| *36* | Shih-Louis | Gaussian | AMH | FGM | Fischer-Kock |
| *37* | Shih-Louis | Gaussian | AMH | FGM | Fischer-Kock |
| *38* | Shih-Louis | Gaussian | AMH | FGM | Fischer-Kock |
| *39* | Shih-Louis | Gaussian | AMH | FGM | Fischer-Kock |
| *40* | Shih-Louis | Gaussian | Fischer-Hinzman | AMH | FGM |
| *41* | Shih-Louis | Gaussian | Fischer-Hinzman | Rafferty | AMH |
| *42* | Shih-Louis | Gaussian | AMH | FGM | Fischer-Kock |
| *43* | Shih-Louis | Gaussian | AMH | FGM | Fischer-Kock |
| *44* | Shih-Louis | Gaussian | AMH | FGM | Fischer-Kock |
| *45* | Shih-Louis | Gaussian | AMH | FGM | Fischer-Kock |
| *46* | Shih-Louis | Gaussian | AMH | FGM | Fischer-Kock |
| *47* | Shih-Louis | Gaussian | AMH | t | FGM |
| *48* | Shih-Louis | Gaussian | AMH | t | FGM |

Table S2. Limits for wave overtopping and structural design of breakwaters, seawalls, dikes, and dams from (Van der Meer et al. 2018). Italicized text represents the design type and associated limits which are most relevant for this study, which is focused vegetated levee crests with relatively small significant wave heights.

| **Design type** | **Mean discharge**  **q (flow rate, L/s/m)** | **Maximum volume**  **Q (total flow, L/m)** |
| --- | --- | --- |
| Rubble mound breakwaters;  H_m0_ > 5 m; no damage | 1 | 2000 – 3000 |
| Rubble mound breakwaters; H_m0_ > 5 m; rear side designed for wave overtopping | 5-10 | 10,000 – 20,000 |
| Grass covered crest and landward slope; maintained and closed grass cover;  H_m0_ = 1 – 3 m | 5 | 2,000 – 3,000 |
| Grass covered crest and landward slope; not maintained grass cover, open spots, moss and bare patches;  H_m0_ = 0.5 – 3 m | .1 | 500 |
| *Grass covered crest and landward slope; H_m0_ < 1 m* | *5 – 10* | *500* |
| Grass covered crest and landward slope; H_m0_ < 0.3 m | No limit | No limit |
|  |  |  |
